# [^18^F]PARPi Imaging Is Not Affected by HPV Status In Vitro

**DOI:** 10.1155/2021/6641397

**Published:** 2021-01-20

**Authors:** Navjot Guru, Paula Demétrio De Souza França, Giacomo Pirovano, Cien Huang, Snehal G. Patel, Thomas Reiner

**Affiliations:** ^1^Department of Radiology, Memorial Sloan Kettering Cancer Center, 1275 York Avenue, New York, New York 10065, USA; ^2^Department of Otorhinolaryngology and Head and Neck Surgery, Federal University of São Paulo, SP, Brazil; ^3^Department of Surgery, Memorial Sloan Kettering Cancer Center, 1275 York Avenue, New York, New York 10065, USA; ^4^Department of Radiology, Weill Cornell Medical College, 1300 York Avenue, New York, New York 10065, USA; ^5^Chemical Biology Program, Memorial Sloan Kettering Cancer Center, 1275 York Avenue, New York, New York 10065, USA

## Abstract

**Background:**

Human papillomavirus- (HPV-) associated oropharyngeal squamous cell carcinomas (OPSCCs) are clinically and pathologically distinct from HPV-negative tumors. Here, we explore whether HPV affects functional biomarkers, including *γ*H2AX, RAD51, and PARP1. Moreover, the role of [^18^F]PARPi as a broadly applicable imaging tool for head and neck carcinomas is investigated.

**Methods:**

HPV-positive and HPV-negative cell lines were used to evaluate the *γ*H2AX, RAD51, and PARP1 expression with immunoblotting and immunofluorescence. Effects of external beam ionizing radiation were investigated *in vitro*, and survival was investigated via colony-formation assay. [^18^F]PARPi uptake experiments were performed on HPV-negative and HPV-positive cell lines to quantify PARP1 expression. PARP1 IHC and *γ*H2AX foci were quantified using patient-derived oropharyngeal tumor specimens.

**Results:**

Differences in DNA repair were detected, showing higher RAD51 and *γ*H2AX expression in HPV-positive cell lines. Clonogenic assays confirm HPV-positive cell lines to be significantly more radiosensitive. PARP1 expression levels were similar, irrespective of HPV status. Consequently, [^18^F]PARPi uptake assays demonstrated that this tracer is internalized in cell lines independently from their HPV status.

**Conclusion:**

The HPV status, often used clinically to stratify patients, did not affect PARP1 levels, suggesting that PARP imaging can be performed in both HPV-positive and HPV-negative patients. This study confirms that the PET imaging agent [^18^F]PARPi could serve as a general clinical tool for oropharyngeal cancer patients.

## 1. Introduction

More than half a million patients were diagnosed with oral or oropharyngeal squamous cell carcinoma (SCC) in the United States in 2019 [1]. Squamous cell carcinomas constitute 90% of the head and neck cancers and are one of the most common cancers worldwide [2]. Although the incidence of head and neck cancers is decreasing in the United States, the rate of oropharyngeal squamous cell carcinoma (OPSCC) increased by 2.5% per year [3]. Oral and oropharyngeal tumors present different risk factors. Although the consumption of tobacco and alcohol are well-established risk factors, they do not explain, for example, the increased incidence of tongue OSCC in young women. Risk factors in those cases have not yet been defined [4]. Risk factors associated with oropharyngeal squamous cell carcinoma, on the other hand, are mainly associated with the infection with human papillomavirus (HPV) [4]. In those cases, usually younger patients in their 40s and 50s are affected [5–7]. Although HPV infection is responsible for approximately 80% of oropharyngeal cancer, tobacco and alcohol consumption are also associated.

The treatment plan for a head and neck cancer patient depends on various factors such as location of the tumor, stage of the disease, and size of the lesion [3]. Moreover, the abovementioned risk factors play a very important role in the management decisions. This is related to two clinical trials, ECOG 2399 and RTOG 0129, conducted in patients harboring SCC of the head and neck (HN), along with several retrospective studies, which demonstrated that HPV-positive OPSCCs have better prognosis and better response to chemo/radiotherapy when compared to HPV-negative patients [8, 9]. The findings prompted the American Joint Committee on Cancer (AJCC) to modify the staging system where now, patients are stratified based on their HPV status [10].

In order to understand the reason for the better outcomes observed on concurrent chemoradiotherapy by the HPV-positive group of patients, studies have attempted to correlate their enhanced radiosensitivity to the underlying mechanisms of DNA repair and cell cycle control [11–16]. In addition, high-risk HPV subtypes, HPV-16 and HPV-18, encode two viral oncoproteins E6 and E7 which disrupt normal host cell division by targeting p53 and the retinoblastoma protein (pRb), respectively [17]. HPV-16 E7 has been shown to induce an increase in DNA double-strand breaks and *γ*H2AX nuclear foci, a DNA damage response marker [17]. Moreover, it has also been correlated with the overexpression of poly (ADP-ribose) polymerase (PARP), a single-strand DNA repair enzyme, and the increased level of RAD51, in response to radiation [18]. Further analysis of these markers through patient samples is performed in this study to understand these cellular mechanisms associated with the better prognosis of HPV-positive cancer.

Considering the importance of imaging in head and neck cancer treatment plans, we have recently published a paper stating the potential of fluorine-18 labeled poly (ADP-ribose) polymerase1 inhibitor ([^18^F]PARPi) as an alternative imaging tool in head and neck carcinomas that can provide high-contrast images compared to [^18^F]fluoro-deoxy-D-glucose ([^18^F]FDG) [19].

[^18^F]FDG is a glucose-mimicking metabolic tracer; therefore, it is retained not only in tumors but also in some normal tissues with high metabolism [20]. In contrast, [^18^F]PARPi is a DNA repair marker; therefore, it accumulates mostly in the nuclei of tumor cells, making it a good tracer for head and neck cancer imaging. PARP1 is a nuclear enzyme that plays an essential role in DNA damage repair. Several previous publications have demonstrated approaches to image PARP1 in vivo; see [21, 22] for an overview. The structure of [^18^F]PARPi is derived from the scaffold of Olaparib, an FDA-approved PARP1 inhibitor widely used as a therapeutic agent in gynecological malignancies [23]. [^18^F]PARPi has been previously shown to bind specifically to tumor areas with a minimal off-target uptake [24, 25].

As the HPV status affects DNA double-strand break repair, we aimed to address if the HPV status had an effect on PARP1 overexpression [26–30] and therefore on [^18^F]PARPi uptake in oral and oropharyngeal cancer. With the present study, we evaluated the diagnostic value of [^18^F]PARPi in oropharyngeal cancer which appears to be independent of a patient's HPV status. However, further *in vivo* and patient data is required to further interrogate this.

## 2. Materials and Methods

### 2.1. Cell Culture and Reagents

All cell lines were grown in a monolayer culture at 37°C in a 5% CO_2_ humidified atmosphere. We cultured three HPV-positive (UPCI:SCC 090, UPCI:SCC 154, and UPCI:SCC 152) and three HPV-negative (FaDu, Cal 27, and SCC 15) cell lines. All media contain 10% (*v*/*v*) fetal bovine serum (FBS) and 1% penicillin and streptomycin. Both UPCI:SCC 090 (ATCC, Manassas, VA) and UPCI:SCC 154 (ATCC, Manassas, VA) cell lines were grown in MEM with 2 mM L-glutamine. The UPCI:SCC 152 (ATCC, Manassas, VA) was grown in MEM with 2 mM of L-glutamine, 50 *μ*g/mL of Gentamicin, and 11.2 mL/L of NEAA. Cal 27 (ATCC, Manassas, VA) cells were cultured in DMEM and the FaDu (ATCC, Manassas, VA) cells in MEM medium. The SCC 15 (ATCC, Manassas, VA) cell line was cultured in a 1 : 1 mixture of high glucose DMEM and F12 medium containing 1.2 g/L of sodium bicarbonate, 2.5 mM L-glutamine, 15 mM HEPES, and 0.5 mM of sodium pyruvate supplemented with 400 ng/mL of hydrocortisone 500 mL solution.

### 2.2. Western Blot

Cell pellets obtained from 80% confluent flasks were washed twice with PBS and lysed with 200 *μ*L ice cold RIPA buffer with Triton X100 (Boston BioProducts, Ashland, MA), one Mini Complete Protease Inhibitor Cocktail tablet (Roche, Indianapolis, IN), and one PhosSTOP tablet (Roche, Indianapolis, IN). Protein quantification was performed using bicinchoninic acid (BCA) protein assay (Pierce, Rockford, IL). For the PARP1 and RAD51 expression, 20 *μ*g of protein lysate was loaded on the gel. For *γ*H2AX, 40 *μ*g of total cellular lysate was loaded on the gel in order to be able to detect the specific signal. Also, the 0.45 *μ*m pore size membrane was replaced with 0.20 *μ*m nitrocellulose membrane (Bio-Rad Laboratories Inc., Germany) for *γ*H2AX detection. The substitution was made because gamma-H2AX is a 17 kDa protein, smaller than all the other proteins detected in this study. During transfer processes, with the 0.45 *μ*m pore size membrane, gamma-H2AX was eluted from the membrane resulting in no detection. Various methods were used to improve detection. The one that enabled detection was the replacement with the 0.20 *μ*m pore size. Transfer time was lowered to 50 min because more extended periods led to the protein being eluted from the membrane. After optimization, 50 min was found to be the optimum time for the gamma-H2AX transfer process. Blocking was performed by 1% BSA in TBS-T solution. Proteins were detected using antibodies specific for PARP1 (1 : 1,000, Invitrogen; PA5-16452), *γ*H2AX (1 : 1000, EMD Millipore; 05-636,) RAD51 (1 : 1000, Abcam; ab63801), *β*-actin (1 : 40,000; Cell Signaling Technology; 3700), and Vinculin (1 : 1000, Abcam; ab129002) with a corresponding horseradish peroxidase- (HRP-) conjugated secondary antibody (1 : 20,000, ab6721(rabbit), ab6789 (mouse), Abcam, USA). Loading control choice was based on the molecular size of the target protein, in order to not interfere with the protein of interest. When a membrane was probed for different proteins, the same loading control was used. Detection was performed using a chemiluminescent substrate (Thermo Scientific #34077, Super Signal West Pico, USA). The bands were visualized using an automated blot processing machine (Ewen-Parker X-ray Corporation, New York, USA) with a light-sensitive clear blue X-ray film (Thermo Scientific, 24 × 30 cm, SB2324231, Belgium) with 1 min exposure time.

### 2.3. Immunofluorescence

4.0‐5.0 × 10^5^ cells from all six cell lines were plated on a 6-well plate. On the following day, the plated cells were irradiated with 2 Gy *γ*-rays in a cesium irradiator (J.L. Shepherd, San Fernando, CA). Before blocking, cells were washed with 300 *μ*L of PBS. Using 3% paraformaldehyde (PFA) in a PBS solution, cells were fixed for 10 min at 2 different time points: 30 min and 24 h. Blocking was performed for at least 1 h in 300 *μ*L PBS with 1% BSA, 0.1% Triton, and 1% Goat serum at room temperature. After blocking, cells were incubated with primary antibodies: *γ*H2AX and RAD51 in 1% BSA for 24 h. After washing with PBS, cells were incubated with Alexa 488 goat anti-rabbit (1 : 1200, Invitrogen; A11070) and Alexa 594 goat anti-mouse (1 : 1200, Invitrogen; A11020) in 1% BSA for 1 h at room temperature in the dark. To mount the cover slip containing cells on the microscope slide, 20 *μ*L of vector shield solution with DAPI (H-1200, Vector Labs) was used to counterstain the nuclei. Using an inverted confocal microscope (LSM 880, Zeiss Confocal, Germany), cells were imaged and the number of RAD51 and *γ*H2AX foci present in the cells was quantified using an ImageJ plugin software. In order to detect foci in patient samples, 0.5 *μ*g of *γ*H2AX antibody and 0.1 *μ*g, 0.2 *μ*g, and 0.4 *μ*g of RAD51 antibody were used. The patient data used in the manuscript was conducted under a protocol approved by the Institutional Review Board (IRB). No human approval was required for this study. IgG control was used to confirm specificity and control for false positive signals of the antibody. The slides were scanned (Pannoramic 250 Flash scanner, 3DHISTECH, Budapest, Hungary) at 40x magnification. Adjacent sections were stained with hematoxylin and eosin (H&E). Regions of interest (ROIs) were drawn on the H&E slides (tumor areas) and transported using CaseViewer (3DHistech, Hungary) to the consecutive slide, where immunofluorescence was carried out in order to analyze correct tumor areas. The images shown are representing one of the tumor areas in whole slide, and the mean number of foci includes foci count in all the tumor areas.

### 2.4. Cell Survival

In order to obtain a sufficient colony count, 800-3200 cells were seeded in 6-well plates in triplicates and exposed to known radiation doses. Cells were irradiated (IR) with 0, 2, 4, and 6 Gy using a cesium irradiator. Cells were allowed to grow for 21 days. After 21 days of incubation, the culture media were removed, and cells were stained in crystal violet (0.5%) in methanol for 2 min. Survival fractions were calculated based on the number of microscopic colonies where a colony is defined as >50 cells. The plating efficiency was calculated as previously reported [31]. The mean number of colonies was obtained from triplicates of three independent biological replicates for each cell line. The clonogenic survival curves fit to a linear-quadratic model were obtained as mentioned previously [31].

### 2.5. PARP1 Immunohistochemistry

Immunohistochemistry (IHC) was performed by the Memorial Sloan Kettering (MSK) Molecular Cytology Core Facility (MCCF) and as previously described by our group [32].

### 2.6. [^18^F]PARPi Uptake Assay

Uptake of [^18^F]PARPi was tested in vitro (3 replicates) as previously described [33]. [^18^F]PARPi was synthesized as reported by our group [19] and obtained from the Radiochemistry and Molecular Imaging Probes Core, MSK. See Supplementary Fig. S1 for details. Briefly, 250k HPV-positive and HPV-negative cells were plated 24 h prior to the experiment (*n* = 3). Media was changed, and 1 h later, 370 kBq/well of [^18^F]PARPi were added to the cells. For blocking, cells were incubated with a 100-fold molar excess of Olaparib 1 h before adding [^18^F]PARPi. Media was removed, and cells were washed with PBS and lysed with 1 M sodium hydroxide (NaOH) after 1 h. The lysate was collected, and uptake was determined by measuring radioactivity on a Wizard^2^ automatic *γ*-counter (PerkinElmer, Boston, MA).

### 2.7. Statistical Analysis

Statistical analysis was performed using GraphPad Prism 8. Unless otherwise stated, data points represent mean values and error bars represent standard deviations of biological replicates. *P* values were calculated using Student's unpaired *t*-test. Statistical significance was determined with alpha = 0.05, and the level of significance for each result was displayed as ^∗^*P* < 0.05, ^∗∗^*P* < 0.01, ^∗∗∗^*P* < 0.001, and ^∗∗∗∗^*P* < 0.0001.

## 3. Results

### 3.1. Immunoblotting of PARP1, RAD51, and *γ*H2AX Levels

**Figure 1:**
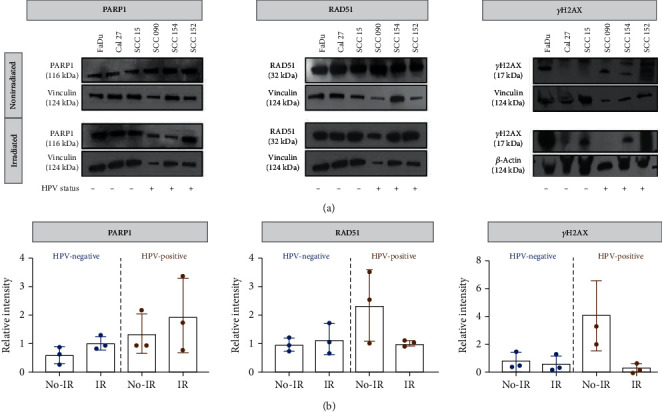
PARP1, RAD51, and *ɣ*H2AX expression in HPV-negative and HPV-positive cell lines. (a) Western blot analysis of three HPV-negative (FaDu, Cal 27, and SCC 15) and three HPV-positive (SCC 090, SCC 154, and SCC 152) cell lines, both with and without external beam irradiation (2 Gy). Vinculin and *β*-actin served as loading controls. For irradiated cell lines, protein extraction was performed 24 h postirradiation. For irradiated PARP1 and RAD51 detection, the same gel was used; therefore, the same loading control is considered. (b) Quantification of PARP1, RAD51, and ɣH2AX from (a). Bars represent grouped mean ± SD of 3 HPV-/+ cell lines.

A panel of cell lines was tested for levels of PARP1, RAD51, and *γ*H2AX (Figure 1(a)). We observed an increase in the expression of PARP1 in both groups of cell lines, HPV-negative (mean ± SD, pre-IR 0.59 ± 0.19, and post-IR 1.0 ± 0.18) and HPV-positive (pre-IR 1.34 ± 1.15 and post-IR 2.0 ± 0.71), after irradiation. No statistically significant results were observed when comparing these two groups of cell lines pre- and postirradiation (Figure 1(b), left panel). Interestingly, although PARP1 levels seemed elevated after irradiation, no such correlation was found when analyzing RAD51 expression (Figure 1(b), middle panel). For HPV-negative cell lines, RAD51 expression did not vary significantly (pre-IR 1.02 ± 0.34 and post-IR 1.20 ± 0.56), and for HPV-positive cell lines after irradiation, a decrease in the expression was observed where the mean ± SD expression for pre-IR was 2.42 ± 1.42, compared to post-IR 1.06 ± 0.24. The level of *γ*H2AX expression in nonirradiated HPV-positive cell lines (4.06 ± 2.52) was higher in HPV-negative cell lines (0.78 ± 0.67) (Figure 1(b), right panel). The mean difference between *γ*H2AX expression in irradiated cell lines decreased due to lower *γ*H2AX levels in both HPV-positive (0.26 ± 0.34) and HPV-negative cell lines (0.61 ± 0.58).

### 3.2. Immunofluorescence Analysis of *γ*H2AX and RAD51 on HPV-Positive and HPV-Negative Cell Lines

**Figure 2:**
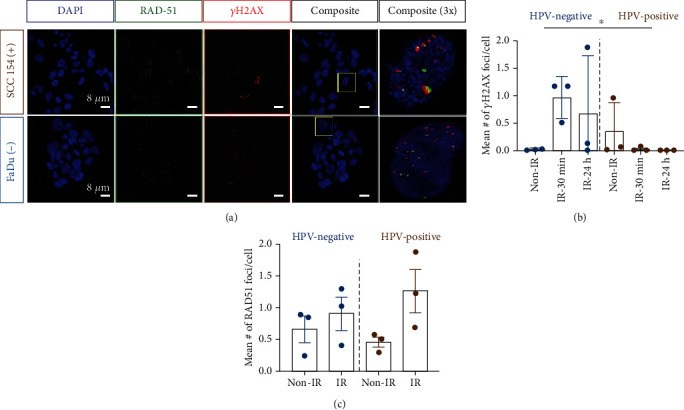
Effect of radiation on HPV-negative and HPV-positive cells. (a) Immunofluorescence staining using ɣH2AX and RAD51 antibodies performed on three HPV-negative (FaDu, Cal 27, and SCC 15) and three HPV-positive (SCC 090, SCC 154, and SCC 152), irradiated (2 Gy) and nonirradiated (0 Gy), cell lines. Representative images showing DAPI (blue), RAD51 (green), and ɣH2AX (red), 24 h postirradiation. IgG control in Supplementary Fig. S3. (b, c) Quantification of the mean number of ɣH2AX and RAD51 foci calculated per cell for different conditions (^∗^*P* < 0.05, Student *t*-test).

In order to visualize and quantify the DNA strand breaks pre- and postirradiation, antibodies against *γ*H2AX and RAD51 were used. The staining was performed on cells that were either nonirradiated or irradiated (30 min and 24 h postradiation) (Figure 2(a), Supplementary Fig. S2A). The mean number of foci was calculated for *γ*H2AX (Figure 2(b)) and RAD51 (Figure 2(c)) to determine the difference in DNA damage in HPV-negative and HPV-positive cell lines. Heterogeneity within HPV-negative and HPV-positive cell lines was observed such as SCC 15 expressing a higher number of *γ*H2AX foci pre- and postirradiation compared to all other cell lines. In HPV-negative cells, we observed an increase in residual double-strand breaks after radiation. We also observed a significantly higher number of gH2AX foci (^∗^*P* < 0.05) at the 30 min postirradiation time point. Besides this, the mean number of the RAD51 focus count increased after irradiation and returned to baseline at 24 h. In HPV-positive cells, the mean number of gH2AX foci was closer to baseline, in both 30 min and 24 h postirradiation, compared to that of HPV-negative cells. Besides this, no difference was detected between pre and 30 min postirradiation in the RAD51 focus numbers. For the 24 h time point, instead of returning to baseline as HPV-negative cells did, the mean number of foci continued to show an increase.

### 3.3. Effect of Irradiation on Survival of HPV-Positive and HPV-Negative Cells

**Figure 3:**
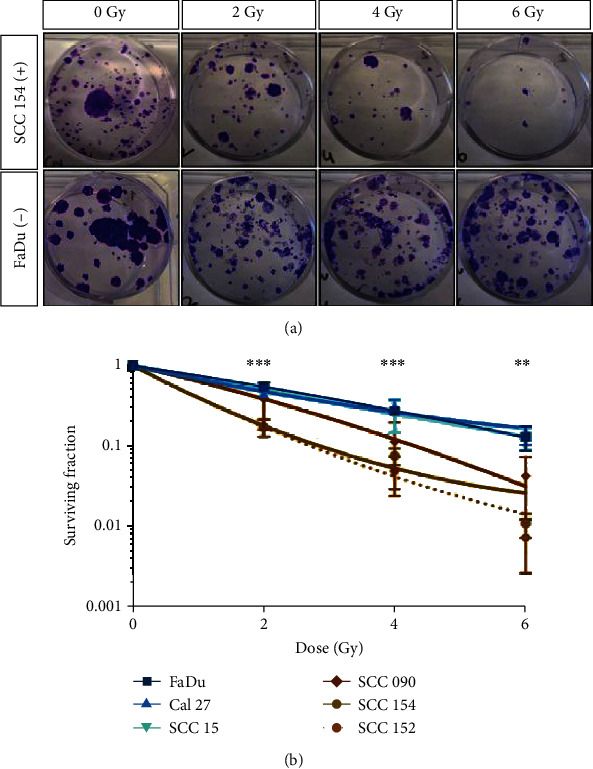
Effect of radiation on HPV-negative and HPV-positive cell survivability. (a) Examples of clonogenic growth observed in HPV-negative, FaDu, and HPV-positive SCC 154 cell lines at 0, 2, 4, and 6 Gy external beam irradiation. (b) Surviving fraction curve of three HPV-negative and three HPV-positive cell lines after 0, 2, 4, and 6 Gy irradiation. Means ± SD of three independent experiments with three replicates each. Statistical significance is shown between FaDu and SCC 154. ^∗∗^*P* < 0.01 and ^∗∗∗^*P* < 0.001. Quantification for all cell lines is shown in Supplementary Fig. S2B.

We evaluated the effect of radiation by performing clonogenic assays on HPV-positive (*n* = 3) and HPV-negative (*n* = 3) cell lines. Corroborating previously published literature [12, 15], we observed that HPV-positive cells are more radiosensitive than HPV-negative cells (Figures 3(a) and 3(b)). Significant differences between the surviving fractions were seen at 2, 4 (^∗∗∗^*P* < 0.001), and 6 Gy (^∗∗^*P* < 0.01) radiation doses in FaDu, an HPV-negative cell line, and SCC 154, an HPV-positive cell line. This difference is also evident by the reduction in colony count. The surviving fraction remains significant at all doses between HPV-negative and HPV-positive cell lines (Figures 3(a) and 3(b), Supplementary Fig. S2B).

### 3.4. PARP1 Expression in Human Oropharyngeal HPV-Negative and HPV-Positive Biospecimens

**Figure 4:**
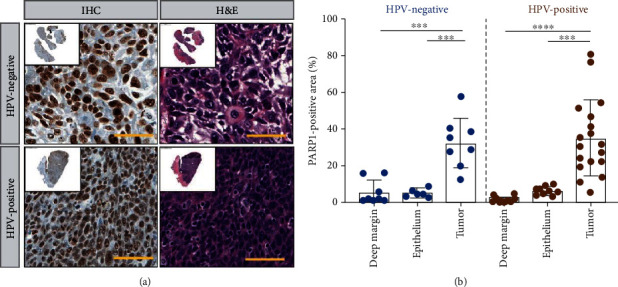
PARP1 expression in patient-derived oropharyngeal biospecimens. (a) PARP1 immunohistochemical staining of HPV-negative and HPV-positive oropharyngeal patient slides and H&E staining of corresponding sections. Scale bar 50 *μ*m. (b) PARP1 quantification of deep margin, epithelium, and tumor for all patient samples (^∗∗∗^*P* < 0.001, ^∗∗∗∗^*P* < 0.0001, Student *t*-test).

In order to investigate the expression of PARP1 in oropharyngeal cancer, HPV-negative (*n* = 7) and HPV-positive (*n* = 18) samples were derived from patients' posttransoral resection of oropharyngeal tumors. None of the patients received presurgical adjuvant treatment. In order to evaluate the expression of PARP1, 3-5 fields of view from three tissue sites were randomly selected, deep margin (submucosa, muscle tissue), epithelium, and tumor, from the H&E and IHC scan of entire tissue for quantification (Figure 4(a)). Automated thresholding was conducted, and the PARP1-positive area was calculated (Figure 4(b)). We observed a statistical difference (^∗∗∗^*P* < 0.001) between both deep margin and epithelium when compared to tumors in both HPV-positive and HPV-negative samples. No statistical significance was found between HPV-negative and HPV-positive tumor areas (32.49 ± 15.05% and 33.04 ± 23.46%, respectively).

### 3.5. Immunofluorescence in Human Biospecimens

**Figure 5:**
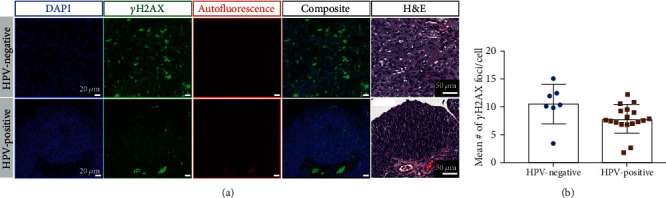
ɣH2AX immunofluorescence in human oropharyngeal biospecimens. (a) Representative immunofluorescence staining images displaying nuclear stain in blue, ɣH2AX in green, and autofluorescence in red. H&E corresponding with the immunofluorescence section is also shown. (b) Quantification of number of focus count per cell in all HPV-negative and HPV-positive oropharyngeal specimens using the entire slide. Each dot represents a patient.

To investigate levels of DSB, *γ*H2AX, a double-strand break marker, was used in HPV-positive and HPV-negative oropharyngeal patient samples. Using H&E as a reference, random tumor sections were chosen, and a focus count was performed (Figure 5(a)). The images in the figure represent one of the tumor areas in the slide. For quantification (mean focus count), all the tumor areas were included. Quantification did not show any statistical difference in the number of *γ*H2AX foci between HPV-negative and HPV-positive patient samples (Figure 5(b)).

### 3.6. [^18^F]PARPi Uptake Assay

**Figure 6:**
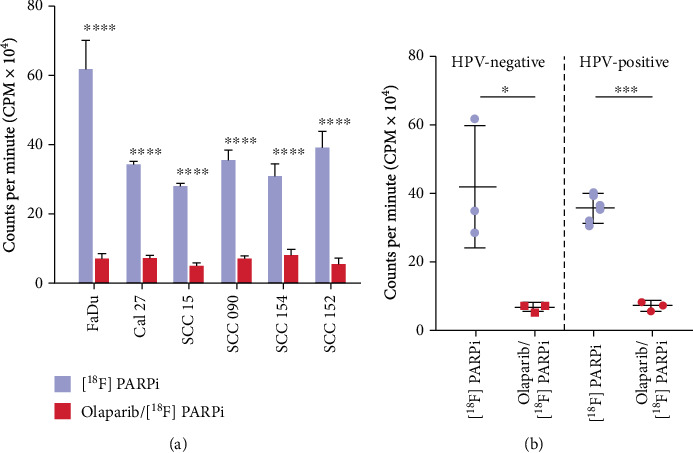
[^18^F]PARPi uptake in HPV-negative and HPV-positive cell lines. (a) *In vitro* internalization assay of 370 kBq of [^18^F]PARPi showing significant specific tracer uptake. Uptake was blocked using 100-fold excess Olaparib 1 h before adding [^18^F]PARPi. (b) Quantification of total uptake in relation to HPV status showing no significant difference of HPV-negative and HPV-positive cell lines (^∗^*P* < 0.05, ^∗∗∗^*P* < 0.001, ^∗∗∗∗^*P* < 0.0001, Student *t*-test).

*In vitro* internalization of [^18^F]PARPi was performed using three HPV-negative and three HPV-positive cell lines. In order to analyze the specificity, a 100-fold excess dose of Olaparib was used to outcompete the tracer uptake. A significant blockable signal was observed in all the cell lines (^∗∗∗∗^*P* < 0.0001) (Figure 6(a)) confirming specificity of the imaging agent. Mean counts per minute calculated between HPV-negative and HPV-positive cell lines did not show any statistically significant difference when compared to each other (Figure 6(b)).

## 4. Discussion

High-risk HPV subtypes, HPV-16 and HPV-18, encode two viral oncoproteins, E6 and E7, which play a role in disrupting normal host cell division by targeting p53 and the retinoblastoma protein (pRb), respectively [17]. HPV-16 E7 has been shown to induce an increase in DNA double-strand breaks and, consequently, in the expression of *γ*H2AX [18]. Moreover, it has also been correlated with the overexpression of PARP1 [17] and RAD51 [18], in response to radiation. For this reason, we speculated that HPV status might affect the uptake of [^18^F]PARPi, a PET imaging agent for head and neck cancers, which binds specifically to PARP. In order to confirm the correlation of the HPV status with cellular and molecular mechanisms underlying favorable prognosis, we investigated *γ*H2AX, RAD51, and PARP1 protein expressions in HPV-positive and HPV-negative cell lines and oropharyngeal carcinoma patient samples.

We observed higher expression levels of double-strand break biomarkers in HPV-positive cell lines compared to those in HPV-negative cell lines, confirming previous findings [18]. In response to radiation, a decline in expression level of both *γ*H2AX and RAD51 was detected. This reduction can be attributed to the intrinsic radiosensitivity of HPV-positive cell lines which was confirmed through clonogenic assays. HPV-positive cell lines displayed significant radiosensitivity at all dose levels which show a higher cell death and a higher probability of getting washed away during the washing steps in immunofluorescence microscopy and western blot. The inconsistency observed in RAD51 levels can be related to the divergent role of RAD51 where, on the one hand, the E7 oncoprotein increases the level of RAD51 protein [18, 34]; on the other hand, p16^INK4A^ plays a role in decreased RAD51 recruitment, thus inhibiting homologous recombination repair [15]. Moreover, a recent study has demonstrated the role of the HPV16 E7 oncoprotein in promoting error-prone microhomology-mediated end-joining while suppressing canonical nonhomologous end-joining (NHEJ) [35]. Therefore, further in-depth studies are required to understand the effect of HPV status on RAD51 expression, to establish a consistent pattern and their relation to radiosensitivity.

Different studies have suggested the persistence of *γ*H2AX foci in HPV-positive cell lines for the favorable prognosis in HPV-associated tumors and their increased radiosensitivity [16, 29]. However, similar results were not observed in this study. Also, patient sample size is a limiting factor; thus, clinical data with a larger sample size can be helpful in understanding the differences seen in HPV-positive and HPV-negative patients in response to therapy.

HPV correlation to single-strand DNA breaks was investigated through PARP expression. In immunoblotting, both HPV-negative and HPV-positive cell lines revealed an increasing trend in the expression of PARP1 in response to radiation. This observation aligns with the literature as radiation increases the formation of DNA breaks, and the expression of PARP1, being a DNA repair enzyme, increases [30, 36]. PARP1 IHC also showed significantly higher expression in tumor regions in both HPV-positive and HPV-negative oropharyngeal samples. Tumor regions displayed significantly higher PARP1 expression compared to deep margin and epithelium. While no significance was observed between tumor regions of the two cohorts, these results allowed us to explore the potential effect on the use of [^18^F]PARPi imaging in oropharyngeal carcinomas.

Our lab recently published a paper which demonstrated [^18^F]PARPi as a potential clinical tool for imaging [19]. There have been pitfalls associated with the [^18^F]FDG PET/CT imaging due to its accumulation not only in the tumor area but also in normal cells [37]. The high physiologic uptake can be problematic in the head and neck region, which has complex anatomy and is intrinsically metabolically active [38, 39]. Considering the importance of imaging in head and neck cancers to design treatment plans, we here have demonstrated that the diagnostic performance of [^18^F]PARPi is probably independent of HPV status. Through [^18^F]PARPi uptake assays, we demonstrated similar internalization in HPV-negative and HPV-positive cell lines. Since HPV-positive tumors have an entirely different prognostic profile compared to HPV-negative tumors, ensuring that [^18^F]PARPi uptake will not be biased towards either cohort was a necessary factor to explore. This study suggests that [^18^F]PARPi is a valuable imaging tool in head and neck tumors with probably no need to stratify patients based on their HPV status. However, new studies, encompassing larger cohort sizes, are necessary to more accurately access the tracer's capability of imaging both HPV-positive and HPV-negative patients. Since the current study was conducted solely *in vitro*, further *in vivo* and human studies are needed to interrogate the biological factors that may impact the uptake pattern and PARP expression.

## 5. Conclusions

We have demonstrated the ability of [^18^F]PARPi to image oropharyngeal tumors regardless of HPV status. Independence of HPV status and PARP allowed internalization of the tracer with no significant variability. Future experiments will focus on evaluating [^18^F]PARPi imaging in patient cohorts to determine its efficacy for imaging oropharyngeal squamous cell carcinoma with greater specificity.

## Data Availability

The data is available on request.

## Supplementary Materials

Supplementary Materials Figure S1: scheme representing [^18^F]PARPi synthesis. Figure S2: effect of external beam irradiation on HPV-negative and HPV-positive cell lines. Figure S3: ɣH2AX immunofluorescence staining IgG control.
