# Supplementary material for: [18F]PARPi Imaging Is Not Affected by HPV Status In Vitro
Source: Mol Imaging. 2021 Jan 20;2021:6641397. doi: 10.1155/2021/6641397 (PMC8205605; doi:10.1155/2021/6641397)
Supplement: Supplementary Materials — Figure S1: scheme representing [18F]PARPi synthesis. Figure S2: effect of external beam irradiation on HPV-negative and HPV-positive cell lines. Figure S3: ɣH2AX immunofluorescence staining IgG control. [file 6641397.f1.pdf]

SUPPLEMENTARY MATERIAL TO:

[^{18}F]PARPi imaging is not affected by HPV status in vitro

Navjot Guru[1], Paula Demétrio de Souza França[1,2], Giacomo Pirovano[1], Cien Huang[1], Snehal G. Patel[3], Thomas Reiner[1,4,5,*]

[1] Department of Radiology, Memorial Sloan Kettering Cancer Center, 1275 York Avenue, New York, New York, 10065, USA

[2] Department of Otorhinolaryngology and Head and Neck Surgery, Federal University of São Paulo, SP, Brazil

[3] Department of Surgery, Memorial Sloan Kettering Cancer Center, 1275 York Avenue, New York, New York, 10065, USA.

[4] Department of Radiology, Weill Cornell Medical College, 1300 York Avenue, New York, New York, 10065, USA

[5] Chemical Biology Program, Memorial Sloan Kettering Cancer Center, 1275 York Avenue, New York, New York, 10065, USA

***Corresponding author:**

Thomas Reiner, Ph.D.

Department of Radiology, MSK

1275 York Avenue

New York, NY 10065

Phone: 646-888-3461

Email: reinert@mskcc.org

Supplementary figures

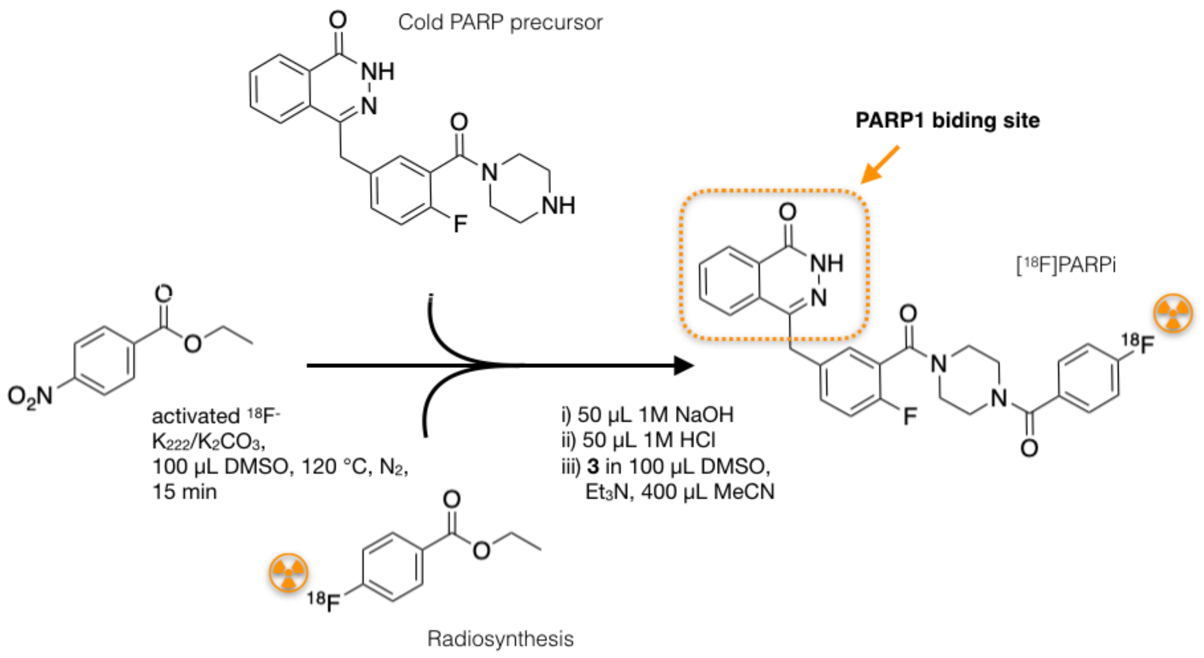

Fig. S1. Scheme representing [^{18}F]PARPi Synthesis. [^{18}F]PARPi can maintain specificity to PARP1 since the chemical modification of the Olaparib scaffold has only limited impact on target binding.

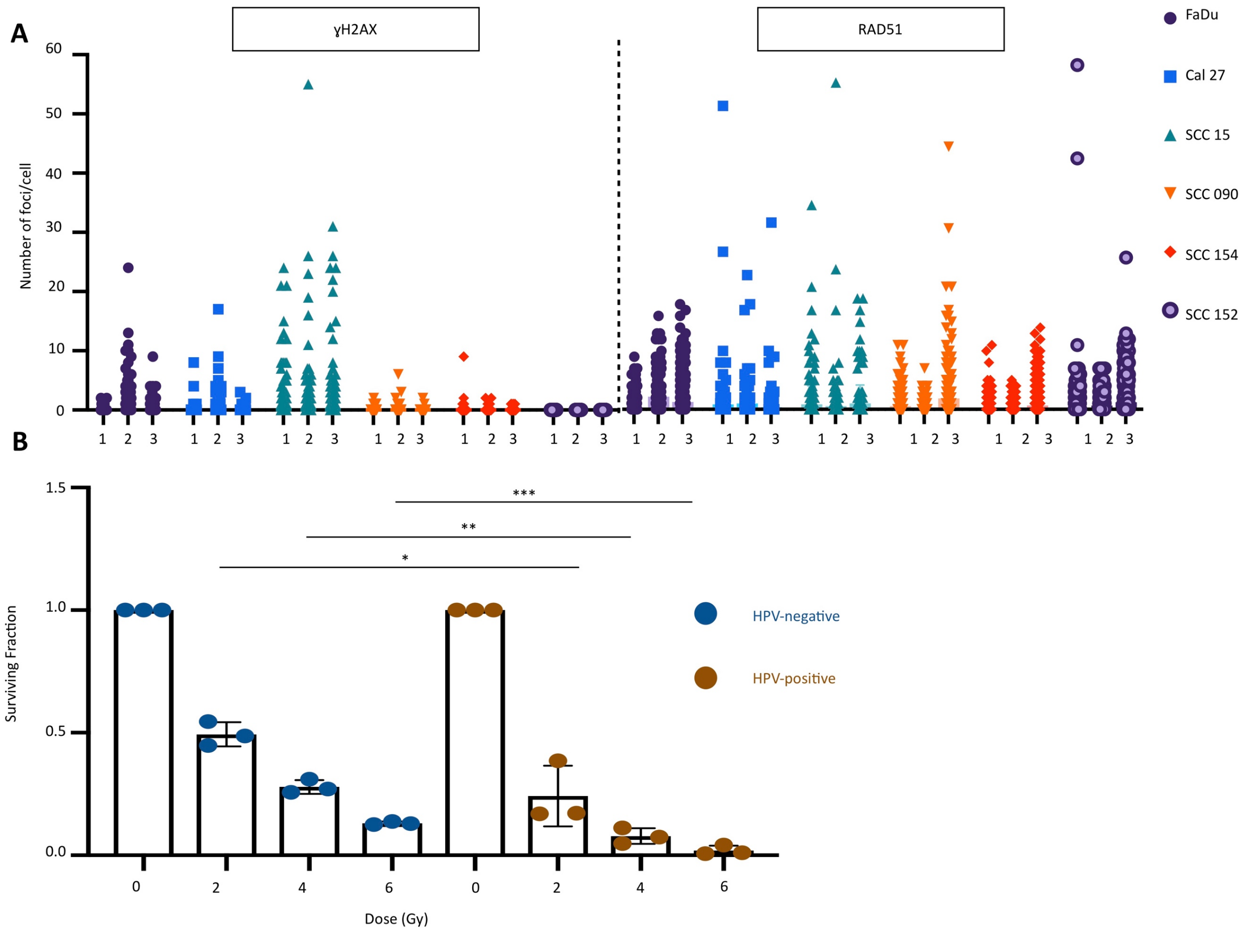

Fig. S2. Effect of external beam irradiation on HPV negative and HPV positive cell lines. (A) Number of foci per cell in individual cell line measured using non-irradiated (0 Gy) and 30 mins and 24 h post irradiation (2 Gy). 1 = Non-irradiated cells, 2 = 30 min post-IR, 3 = 24 h post-IR. **(B)** Surviving fraction analysis of three HPV-negative and three HPV-positive cell lines after 0, 2, 4, and 6 Gy irradiation through clonogenic assay. Each dot represents a cell line. Mean ± SD of three independent experiments with three replicates each (**p* < 0.05, ***p* < 0.01, ****p* < 0.001, student *t*-test).

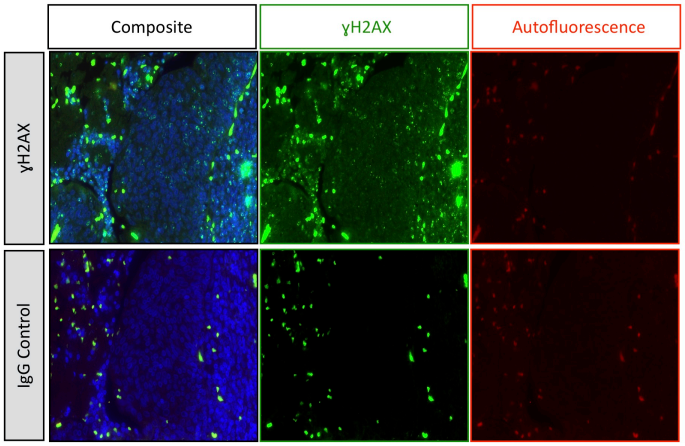

Fig. S3. ɣH2AX Immunofluorescence staining IgG control. Top lane shows patient biospecimen with ɣH2AX staining (0.5µg) and visible foci in green. Bottom lane represents the IgG control where no visible foci is observed. The visible color corresponds to autofluorescence as shown in red which is originated from blood cells.
